# Protein Diversity and Immune Specificity of Hemocyanin From Shrimp *Litopenaeus vannamei*

**DOI:** 10.3389/fimmu.2021.772091

**Published:** 2021-12-07

**Authors:** Xianliang Zhao, Jie Qiao, Pei Zhang, Zehui Zhang, Jude Juventus Aweya, Xiaohan Chen, Yongzhen Zhao, Yueling Zhang

**Affiliations:** ^1^ Institute of Marine Sciences and Guangdong Provincial Key Laboratory of Marine Biotechnology, Shantou University, Shantou, China; ^2^ College of Fisheries, Henan Normal University, Xinxiang, China; ^3^ Guangxi Key Laboratory of Aquatic Genetic Breeding and Healthy Aquaculture, Guangxi Academy of Fishery Sciences, Nanning, China; ^4^ Southern Marine Science and Engineering Guangdong Laboratory, Guangzhou, China

**Keywords:** *Litopenaseus vannamei*, hemocyanin, proteomics approach, diversity, pathogen recognition, glycosylation

## Abstract

Hemocyanin is an important non-specific innate immune defense molecule with phenoloxidase, antiviral, antibacterial, hemolytic, and antitumor activities. To better understand the mechanism of functional diversity, proteomics approach was applied to characterize hemocyanin (HMC) expression profiles from *Litopenaeus vannamei*. At first, hemocyanin was purified by Sephadex G-100 and DEAE-cellulose (DE-52) columns from shrimp serum, and 34 protein spots were identified as HMC on the 2-DE gels. Furthermore, we found that 9 HMC spots about 75 or 77 kDa were regulated by *Streptococcus agalactiae* and *Vibrio parahaemolyticus* infection at 6, 12, and 24 h. In addition, 6 different pathogen-binding HMC fractions, viz., HMC-Mix, HMC-Vp, HMC-Va, HMC-Vf, HMC-Ec, and HMC-Sa, showed different agglutinative and antibacterial activities. Moreover, lectin-blotting analysis showed significant differences in glycosylation level among HMC isomers and bacteria-binding HMC fractions. Particularly, the agglutinative activities of the HMC fractions were almost completely abolished when HMC was deglycosylated by O-glycosidase, which suggest that O-linked sugar chains of HMC played important roles in the innate immune recognition. Our findings demonstrated for the first time that *L. vannamei* HMC had molecular diversity in protein level, which is closely associated with its ability to recognize diverse pathogens, whereas glycan modification probably contributed to HMC’s diversity and multiple immune activities.

## 1 Introduction

Hemocyanin (HMC) is a large copper-containing respiratory protein found in the hemolymph of mollusks and arthropods. Initially, the immune significance of HMC was dismissed until the late 1990s when Decker and Rimke demonstrated unequivocally its phenoloxidase activity (1). Further research revealed that HMC may be a novel and important non-specific innate immune defense molecule (2, 3). It has been reported that HMC could be functionally converted into phenoloxidase (4) antimicrobial peptides (5–8), antiviral (9–12), antitumor agent (13–17), antifungal (18), agglutinin (19, 20), hemolysin (21), and regulatory proteins (22, 23). Moreover, our previous findings indicated that HMC from shrimp *Litopenaeus vannamei* reacted with human Ig as an antigen (5, 19, 24) and acted as an immune-enhancing protein (25, 26). These results show HMC’s roles in many immune activities and its importance in invertebrate innate immune system. However, so far, little is known about the molecular mechanism of HMC’s functional diversity in *L. vannamei*.

Despite the long belief that invertebrates lacked an adaptive system and possessed simple, innate immune systems, recent evidence suggests that diversification of immune-response proteins against pathogens and high immune specificities are also found in invertebrates (27–29). Interestingly, our recent observations and those of others indicated that *L. vannamei* HMC also displayed high molecular diversity, including single-nucleotide polymorphisms (SNPs) and different types of variants, both of which could be modulated by pathogenic infection (30–35), suggesting that HMC functional diversity may be associated with its molecular polymorphism. However, the diversity of HMC at the protein level has not been studied so far.

In this study, we demonstrated *via* proteomic approach the diversity of HMC at the protein level in *L. vannamei*. Furthermore, the association between polymorphism at the protein level and resistance to diverse pathogens, and the mechanisms were also characterized. These findings will be helpful for understanding the molecular basis of HMC multifunctionality and establishing the novel strategies for shrimp disease control.

## 2 Materials and Methods

### 2.1 Animal and Preparation of Shrimp Serum

Healthy penaeid shrimps (*L. vannamei*) with approximate size of 10–14 cm in length and 15–20 g in weight from Shantou Huaxun Aquatic Product Corporation were obtained and reared in 25-L seawater tanks at 25°C. Air was continuously supplied using an electric pump.

Hemolymph was taken directly from the pericardial sinus using a sterile tube and then allowed to clot overnight at 4°C. The serum was separated after centrifuging at 3,000 *g* for 20 min and kept at −20°C until analysis. All animal experiments were conducted in accordance with the recommendations set forth in the Animal Ethics Procedures and Guidelines of the People’s Republic of China.

### 2.2 Purification of HMC by Chromatographies

HMC purification was performed by gel-filtration chromatography and anion-exchange chromatography as previously described with some modifications (5). Briefly, 2 ml of *L. vannamei* serum was loaded onto a Sephadex G-100 column, and then, the column was washed with Tris–HCl buffer (0.05 M, pH 8.0) at a flowrate of 1 ml/min until absorbance at 280 nm reached baseline. The eluted proteins were concentrated by ultrafiltration centrifuge tube. Then, the concentrated proteins (8 ml) were loaded onto a DEAE-cellulose (DE-52) column, equilibrated with 0.01 M pH 7.5 phosphate-buffered saline (PBS) buffer. Elution was performed with 0.2 M NaCl at a flowrate of 1 ml/min. Eluted proteins from the main peak (potential HMC) was determined by a modified Bradford assay (Bio-Rad, USA) and stored at −20°C until analysis.

### 2.3 One-Dimensional Sodium Sulfate–Polyacrylamide Gel Electrophoresis and Its Immunoblotting

Identification of the potential HMC was carried out using one**-**dimensional sodium sulfate–polyacrylamide gel electrophoresis (1-DE) and its immunoblotting. In brief, 1-DE was performed using a 5% stacking gel (pH 6.8) and a 10% separating gel (pH 8.9) in Tris–glycine buffer (pH 8.3). Then, the gel was transferred to a polyvinylidene fluoride (PVDF) membrane with a semi-dry transfer apparatus according to the manufacturer’s instructions. The membrane was blocked for 1 h with 5% skim milk in Tris-buffered saline (TBS) (20 mM Tris, 0.15 M NaCl, pH 7.4) at room temperature, then incubated with rabbit anti-shrimp HMC antisera (1:1,000 dilution) and goat anti-rabbit immunoglobulin G (IgG)–horseradish peroxidase (HRP) (1:3,000 dilution) antibodies at room temperature for 40 min and 1 h, respectively. Finally, the membrane was washed and developed with substrate (3′3-diminobenzidine, DAB) until optimum color developed.

### 2.4 Two-Dimensional Polyacrylamide Gel Electrophoresis and its Immunoblotting

To investigate the diversity of HMC, two-dimensional polyacrylamide gel electrophoresis (2-DE) and its immunoblotting were performed as our previous descriptions with some modifications (36). Briefly, a total of 30 µg of HMC in rehydration buffer [containing 7 M urea, 2 M thiourea, 4% CHAPS, 65 mM dithiothreitol (DTT), 0.2% bio-lyte, and 0.001% bromophenol blue, pH 3–10] was used to rehydrate the IPG strip (7 cm, pH 4.7–5.9 Bio-Rad, Hercules, CA) for 12 h. The isoelectric focusing (IEF) was performed at a constant temperature of 20°C using a continuous increase in voltage (up to 4,000 V) until reaching 35,000 Vh. Prior to the second dimension, the focused IPG was incubated for 15 min in an equilibration buffer containing 20% w/v glycerol, 2% sodium dodecyl sulfate (SDS), 0.375 M Tris–HCl (pH 8.8), 2% DTT, then further equilibrated for 15 min in a similar buffer in which 2% DTT was replaced with 2.5% of iodoacetamide. The strip was placed onto the top of a 12% 1-DE gel. Low-melting point agarose was used to cover the IPG strip and filter paper. Separation of proteins was carried out in the same conditions as described above for 1-DE. Following 2-DE, immunoblotting analysis was carried out as the same descriptions in **Section 2.3** for the identification of HMC. Gels were stained with colloidal Coomassie, and spot volumes were compared using the image analysis software PDQuest Analysis Software (Bio-Rad).

### 2.5 MALDI-TOF-TOF Mass Spectrometry

To identify the proteins reacted with rabbit anti-shrimp HMC antibodies in the 2-DE map, matrix-assisted laser desorption ionization–time of flight–time of flight (MALDI-TOF-TOF) mass spectrometry analysis was further performed as our previous descriptions (26). The spots were excised from 2-DE gels, and the gel plug was digested with trypsin; then, 0.5 µl of the peptide mixture was mixed with the matrix a-cyano-4-hydroxycinnamic acid (1:1) and spotted onto a stainless steel MALDI plate. MS spectra were obtained using the ABI 4700 Proteomics Analyzer MALDI-TOF-TOF mass spectrometer (Applied Biosystems, Foster City, CA) operating in a result-dependent acquisition mode. Peptide mass maps were acquired in reflection mode (1-keV accelerating voltage) with 1,000 laser shots per spectrum. Six external standards (mass standard kit for the 4700 Proteomics Analyzer calibration mixture, Part Number 4333604, Applied Biosystems, Foster City, CA) were used to calibrate each spectrum to a mass accuracy within 50 ppm. Selected peptide masses were submitted to Mascot (http://www.matrixscience.com/cgi/search_form.pl?FORMVER=2&SEARCH=PMF) for NCBInr databases search.

### 2.6 Pathogens Challenge Tests

For the pathogens challenge tests, two representative bacterial, namely, *Streptococcus agalactiae* (Gram-positive bacterium) and *Vibrio parahaemolyticus* (Gram-negative bacterium) were selected. The shrimps were inoculated intramuscularly by using 1-ml syringes in the second abdominal segment with 50 μl of bacterial inoculum (10^7^–10^8^ CFU/ml). The treated animals were then returned to the tanks at room temperature. Hemolymph was collected at 0, 6, 12, and 24 hpi. The following procedures for serum preparation and 2-DE analysis were performed as described in **Sections 2.1** and **2.4**.

### 2.7 Bacterial Pull-Down Assay

To purify the HMC fractions binding with pathogens, bacterial pull-down assay was performed as described previously (37). In brief, five kinds of strains including *S. agalactiae*, *V. parahaemolyticus*, *Vibrio alginolyticus*, *Vibrio fluvialis*, and *Escherichia coli* K12 were used for incubation with *L. vannamei* serum *in vitro*. The bacterial cells were harvested at OD_600_ of 1.0 and washed three times with 0.9% (w/v) saline. Then, the supernatant was boiled for 10 min and harvested by centrifugation at 5,000 *g* for 10 min. Three hundred microliters of serum was mixed with 300 µl bacterial solution (1.0 × 10^9^ CFU/ml), incubated at room temperature for 2 h, and the sample was centrifuged at 5,000 *g* for 10 min at 4°C. The pellet was washed three times with 0.9% saline and then resuspended in 1 M Tris–HCl (pH 8.0) containing 0.8 M NaCl at 37°C for 2 h. The supernatant incubated with *S. agalactiae*, *V. parahaemolyticus*, *V. alginolyticus*, *V. fluvialis*, and *E. coli* K12, and mixed five bacteria were collected and named as HMC-Sa, HMC-Vp, HMC-Va, HMC-Vf, HMC-Ec, and HMC-Mix, respectively. After determination with a modified Bradford assay (Bio-Rad, USA), these HMC isomers were identified by 1-DE, 2-DE, and their immunoblotting as described in **Sections 2.3** and **2.4**.

### 2.8 Agglutination Assays

Agglutinative activities of six kinds of pathogens-binding HMC fractions, namely, HMC-Sa, HMC-Vp, HMC-Va, HMC-Vf, HMC-Ec, and HMC-Mix, were performed as our previous descriptions (38). Briefly, these bacteria were cultured in broth medium or Luria–Bertani medium overnight at 28 or 37°C (*E. coli* K12). The cells were harvested, washed, and diluted to 10^8^ CFU/ml in TBS-Ca^2+^ (0.05 M Tris, 0.75% NaCl, 0.05 M CaCl_2_). Agglutination tests using the five bacteria and six specimens were performed at 37°C for 30 min. The proteins were twofold diluted in TBS-Ca^2+^ and added into 10 μl of each bacterium. Agglutination was observed in a light microscope and scored as positive (+) or negative (−) compared to a control placing the corresponding bacteria only in the TBS-Ca^2+^ buffer. Agglutinative titer was defined as the highest dilution of the test samples when the agglutination was appeared.

### 2.9 Antibacterial Activity Assays

Antibacterial activity was assessed by the numbers of bacterial colonies grown on the Petri dish according to our previous descriptions (5). *V. alginolyticus* and *V. fluvialis* were cultured in broth medium at 28°C for 12–24 h, then diluted to 10^3^–10^4^ CFU/ml with sterile PBS (0.01 M, pH 7.4). One hundred microliters of bacterial suspension was taken out and mixed with an equal volume of filtered HMC-Sa, HMC-Vp, HMC-Va, HMC-Vf, HMC-Ec, and HMC-Mix (experimental groups) or sterile 0.01 M pH 7.4 PBS (control group) at 37°C for 2 h. Fifty microliters of mixed solution was taken out and incubated on solid broth medium by the spread plate method at 37°C for 12 h, then counted the colony of the experiment group (A_1_) and control group (A_0_). The antibacterial activity was calculated as follows: antibacterial rate = (A_0_ − A_1_)/A_0_ × 100%. All samples were prepared in triplicate, and digital photomicrographs were taken with an Olympus BH-2 microscope.

### 2.10 Lectin-Blotting Analysis

Lectin-blotting analysis of HMC purified by gel-filtration chromatography and anion-exchange chromatography and the six kinds of pathogens-binding HMC fractions was carried out as previously described with some modifications (8). For 1D or 2D lectin blotting, 30 µg of HMC was separated by 1-DE or 2-DE and then transferred to a PVDF membrane as above descriptions in **Sections 2.3** and **2.4**. The membrane was blocked for 2.5 h with 5% bovine serum albumin (BSA) in TBS (20 mM Tris, 0.5 M NaCl, pH 7.4) at room temperature, then incubated with biotinylated lectin, viz., 1:1,000 dilution of concanavalin A (ConA), 1:100 dilution of peanut agglutinin (PNA), 1:500 dilution of ulex europaeus agglutinin (UEA), or 1:1,000 dilution dolichos biflorus agglutinin (DBA), for 1 h and avidin-peroxidase for 40 min at 37°C, respectively. Finally, the membrane was washed and developed with substrate (3′3-diminobenzidine, DAB) until optimum color developed. For Dot-lectin blotting, 1.5 μl of the six kinds of pathogens-binding HMC fractions (0.025, 0.05, or 0.5 mg/ml) was spotted onto a nitrocellulose (NC) membrane, which has been cut into desired size, soaked in TBS for 5 min, and then allowed to dry, using a regulatory pipe, respectively. After drying, the NC membrane was blocked with 2% polyvinylpyrrolidone (PVP, Sigma) for 1.5 h at room temperature. The following procedures for incubating with four biotinylated lectins and avidin-peroxidase and stained with DAB were performed as above described.

### 2.11 Total Glycan Measurement

The carbohydrate content measurement of HMC-Va, HMC-Vf, and HMC-Mix were determined by the colorimetric method as previously described with some modification (5). Briefly, 0, 100, 200, 300, and 400 μl of 0.01 M standard glucose solution were added to tubes; then, the duplicate distillated water was complemented to the final solution volume of 500 μl. Three hundred microliters of 6% (m/v) phenol and 1.5 ml sulfuric acid were added rapidly. After incubation at room temperature for 25 min, the absorbance was detected at 490 nm. Standard curve was constructed using sugar content as ordinate and the absorbance as abscissa. The total glycan content of samples were calculated according to the standard curve after detecting the absorbance as above using 200 μl of 250 μg/ml HMC-Va, HMC-Vf, and HMC-Mix, respectively.

### 2.12 Deglycosylation Assay

Deglycosylation of two HMC fractions was performed with O-glycosidase or N-glycosidase (New England Biolabs, USA) according to the manufacturer’s instructions and previous description (38). Briefly, HMC-Vp or HMC-Vf was deglycosylated under the following conditions: 5 μl of 10× G7 buffer, 3 μl of O-glycosidase or N-glycosidase, 5 μl of neuraminidase (only for O-glycosidase), then added HMC fractions (300 μg/ml for each) up to 50 μl; the reaction solution above was incubated 37°C for 4 h, respectively. The deglycosylated HMC fractions (HMC-Vp or HMC-Vf) by O-glycosidase and N-glycosidase were collected and named as dO-HMC-Vp and dO-HMC-Vf, and dN-HMC-Vp and dN-HMC-Vf, respectively. Deglycosylated HMC fractions (non-treated HMC fractions as control) were further processed by agglutinative activity assays as above described in **Section 2.8**.

### 2.13 Statistical Analysis

In this study, data are presented as mean ± standard deviation (SD). Statistical significance across groups was analyzed using one-way ANOVA analysis; differences were considered to be significant at *p* < 0.05 and extremely significant at *p* < 0.01.

## 3 Results

### 3.1 Protein Diversity of *L. vannamei* HMC

**Figure 1:**
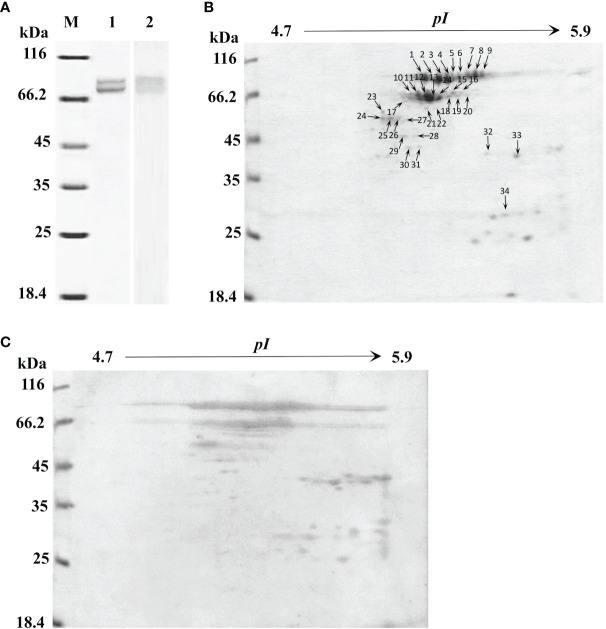
One-dimensional sodium sulfate-polyacrylamide gel electrophoresis (1-DE), two-dimensional polyacrylamide gel electrophoresis (2-DE) and immunoblotting analysis of *L.* hemocyanin. **(A)** 1-DE image of *L. vannamei* hemocyanin purified by gel-filtration chromatography and anion-exchange chromatography (lane 1) and its immunoblotting analysis (lane 2). M, molecular mass markers. Western blotting was performed using rabbit anti-shrimp HMC antisera (1:1,000 dilution) and goat anti-rabbit IgG-HRP (1: 3,000 dilution) antibodies. **(B)** 2-DE image of 30 µg hemocyanin from **(A)** with pH 4.7–5.9 IPG strips. The spots were excised from 2-DE gels, and submitted to protein identification by MALDI-TOF-TOF-MS analysis, the spots marked with number indicated that the HMC proteins identified. **(C)** Immunoblotting was carried out as the same descriptions with **(A)**.

For a global assessment of HMC diversity in shrimp, HMC was first isolated from the hemolymph of *L. vannamei* by gel-filtration chromatography and anion-exchange chromatography and then identified with 1-DE and immunoblotting. As shown in **Figure 1A**, two bands at molecular weights approximately 75 and 77 kDa could react specifically with anti-shrimp HMC antibodies, suggesting that a good separation of the HMC has been achieved. Then, the purified HMC was analyzed by 2-DE (pH 4.7–5.9) and immunoblotting. The pattern of spots obtained from purified HMC is shown in **Figure 1B**. Over 40 distinct protein spots (mainly ranging from 25 to 77 kDa) were detected in 2-DE gels after Coomassie blue staining followed by analysis with the PDQuest software version 8.0. Immunoblotting showed a similar protein profile, indicating that all protein spots were HMC (**Figure 1C**). Furthermore, these spots were excised from gels and subjected to MALDI-TOF-TOF analysis. In total, 34 spots were successfully identified as *L. vannamei* HMC (**Table 1**), of which 6 spots (spots 1–6) and 3 spots (spots 7–9) were confirmed as HMC subunit with 75 and 77 kDa, respectively. Notably, 25 spots (spots 10–34), ranging from 25 to 66 kDa, also showed homology with HMC, suggesting that these may be HMC fragments or modified proteins. These results indicate that *L. vannamei* HMC might possess high molecular diversity at the protein level.

**Table 1:** Identification of proteins from HMC by MALDI-TOF-TOF-MS.

Protein	Accession Name	Description	Species	MW (Da)	*pI*	Expect	Score
1	gi|854403	Hemocyanin	*L. vannamei*	74,934	5.27	7.1e−16	224
2	gi|854403	Hemocyanin	*L. vannamei*	74,934	5.27	3.6e−13	197
3	gi|854403	Hemocyanin	*L. vannamei*	74,934	5.27	2.8e−24	308
4	gi|854403	Hemocyanin	*L. vannamei*	74,934	5.27	5.7e−16	225
5	gi|854403	Hemocyanin	*L. vannamei*	74,934	5.27	3.6e−15	217
6	gi|854403	Hemocyanin	*L. vannamei*	74,934	5.27	1.8e−13	200
7	gi|7414468	Hemocyanin	*L. vannamei*	76,455	5.54	0.82	73
8	gi|7414468	Hemocyanin	*L. vannamei*	76,455	5.54	9e−13	193
9	gi|7414468	Hemocyanin	*L. vannamei*	76,455	5.54	3.6e−10	167
10	gi|854403	Hemocyanin	*L. vannamei*	74,934	5.27	0.0057	85
11	gi|7414468	Hemocyanin	*L. vannamei*	76,455	5.54	15	51
12	gi|7414468	Hemocyanin	*L. vannamei*	76,455	5.54	0.6	65
13	gi|854403	Hemocyanin	*L. vannamei*	74,934	5.27	0.00088	93
14	gi|854403	Hemocyanin	*L. vannamei*	74,934	5.27	0.075	74
15	gi|7414468	Hemocyanin	*L. vannamei*	76,455	5.54	3.4e+03	27
16	gi|854403	Hemocyanin	*L. vannamei*	74,934	5.27	0.026	78
17	gi|854403	Hemocyanin	*L. vannamei*	74,934	5.27	8.9e−06	113
18	gi|854403	Hemocyanin	*L. vannamei*	74,934	5.27	0.068	74
19	gi|854403	Hemocyanin	*L. vannamei*	74,934	5.27	0.91	63
20	gi|854403	Hemocyanin	*L. vannamei*	74,934	5.27	0.012	82
21	gi|854403	Hemocyanin	*L. vannamei*	74,934	5.27	8.9e−09	143
22	gi|7414468	Hemocyanin	*L. vannamei*	76,455	5.54	0.0088	83
23	gi|854403	Hemocyanin	*L. vannamei*	74,934	5.27	0.0056	85
24	gi|7414468	Hemocyanin	*L.vannamei*	76,455	5.54	6.1e+02	35
25	gi|854403	Hemocyanin	*L. vannamei*	74,934	5.27	0.034	77
26	gi|854403	Hemocyanin	*L. vannamei*	74,934	5.27	0.005	86
27	gi|7414468	Hemocyanin	*L. vannamei*	76,455	5.54	2.6	58
28	gi|7414468	Hemocyanin	*L. vannamei*	76,455	5.54	0.015	81
29	gi|854403	Hemocyanin	*L. vannamei*	74,934	5.27	5.5	55
30	gi|854403	Hemocyanin	*L. vannamei*	74,934	5.27	0.00043	96
31	gi|7414468	Hemocyanin	*L. vannamei*	76,455	5.54	2.1e+02	39
32	gi|854403	Hemocyanin	*L. vannamei*	74,934	5.27	1.5e+02	41
33	gi|854403	Hemocyanin	*L. vannamei*	74,934	5.27	5.1	55
34	gi|854403	Hemocyanin	*L. vannamei*	74,934	5.27	2.4e+02	39
a^*^	gi|7414468	Hemocyanin	*L. vannamei*	76,455	5.54	0.00084	101
b^*^	gi|854403	Hemocyanin	*L. vannamei*	74,934	5.27	4.2e−09	154

*Protein band identification in **Figure 3A**.

### 3.2 Responses of HMC Isomers to Pathogen Challenge *In Vivo*

**Figure 2:**
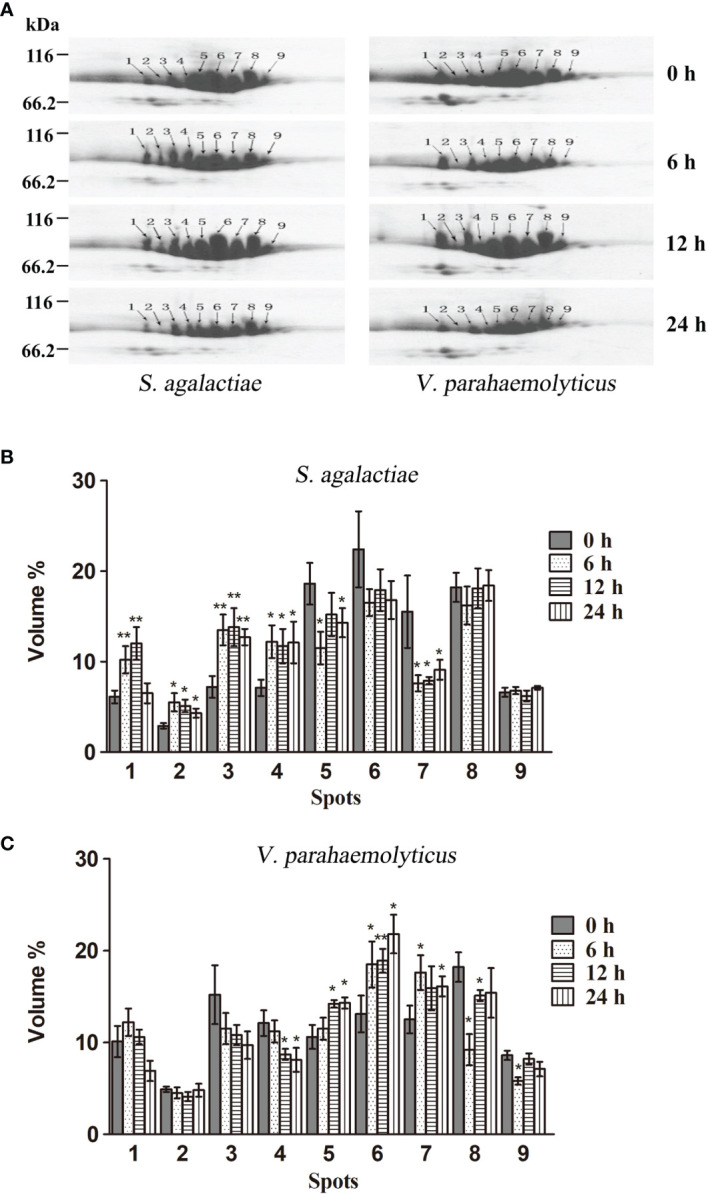
2-DE analysis of the hemocyanin from *L. vannamei* infected with pathogenic bacteria. **(A)** 2-DE analysis of 30 µg hemocyanin from *L. vannamei* treated with *S. agalactiae* (left) and *V. parahaemolyticus* (right) for 0–24 h; 0 h samples were used as control. **(B, C)** The expression level is normalized by densitometry analysis using the image analysis software PDQuest Analysis Software (Bio-Rad); 0 h samples were used as control. *p < 0.05, **p < 0.01.

In order to examine whether different HMC isomers responded to pathogen stimulation, 2-DE analysis was carried out to compare the variation in the nine protein spots in HMC of *L. vannamei* treated with *S. agalactiae* or *V. parahaemolyticus.* As shown in **Figure 2A**, the protein expression of HMC spots increased or decreased to some extent at different time points during the 24-h time period. After treatment with *S. agalactiae* for 6–24 h, spots 1–4 were upregulated, while spots 5 and 7 were downregulated compared with 0 h control group. However, spots 5–7 were upregulated after treatment with *V. parahemolyticus* for 6–24 h, while spots 4, 8, and 9 were downregulated at least at one time point post *V. parahemolyticus* treatment compared with the 0-h control group. Notably, a comparative analysis of the two gels indicated that spot 2 was upregulated about 2.5-, 2.5-, and 1.9-fold after stimulation with *S. agalactiae* at 6, 12, and 24 h, respectively, whereas no significant difference was observed in spot 2 during the entire 24-h period of infection by *V. parahemolyticus* (**Figures 2B, C**). Thus, these results indicated that *L. vannamei* HMC diversity might be related to shrimp’s resistance to specific bacteria *in vivo*.

### 3.3 Agglutinative and Antibacterial Activities of HMC Isomers Bound to Pathogens *In Vitro*

**Figure 3:**
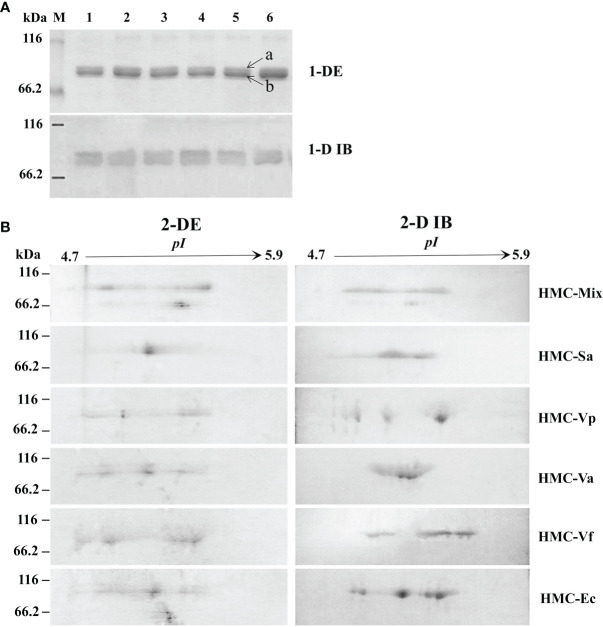
Proteins diversity analysis of six bacteria-binding hemocyanin fractions from *L. vannamei*. **(A)** 1-DE and 1-D immunoblotting analysis of six bacteria-binding hemocyanin fractions; 1–6, HMC-Mix, HMC-Sa, HMC-Vp, HMC-Va, HMC-Vf, and HMC-Ec, respectively. 1-D IB, 1-D immunoblotting. Bands a and b were identified by MALDI-TOF-TOF-MS. Western blotting was performed using rabbit anti-shrimp HMC antisera (1:1,000 dilution) and goat anti-rabbit IgG-HRP (1: 3,000 dilution) antibodies. **(B)** 2-DE and 2-D immunoblotting analysis of six bacteria-binding hemocyanin fractions, i.e., HMC-Mix, HMC-Sa, HMC-Vp, HMC-Va, HMC-Vf, and HMC-Ec. 2-D IB, 2-D immunoblotting. Western blotting was carried out as the same descriptions with **(A)**.

To further determine the relationship between HMC isomers and its resistance to different pathogens *in vitro*, six HMC fractions bound directly to mixed bacteria (HMC-Mix), *S. agalactiae* (HMC-Sa), *V. parahaemolyticus* (HMC-Vp), *V. alginolyticus* (HMC-Va), *V. fluvialis* (HMC-Vf), and *E. coli* K12 (HMC-Ec) were purified by pull-down. Results of the 1-DE and 1-D immunoblotting (1-D IB) showed two similar bands, which were identified as 77 and 75 kDa HMC (**Figure 3A** and **Table 1**). Comparative analysis of 2-DE and 2-D immunoblotting (2-D IB) profiles indicated that there were significant differences in HMC combined with different pathogenic bacteria. For example, the protein spots of HMC-Sa were mainly concentrated in the middle, while HMC-Vp and HMC-Vf were the opposite (**Figure 3B**). Thus, these results indicated that *L. vannamei* HMC diversity might be related to shrimp’s resistance to specific bacteria *in vivo*.

**Figure 4:**
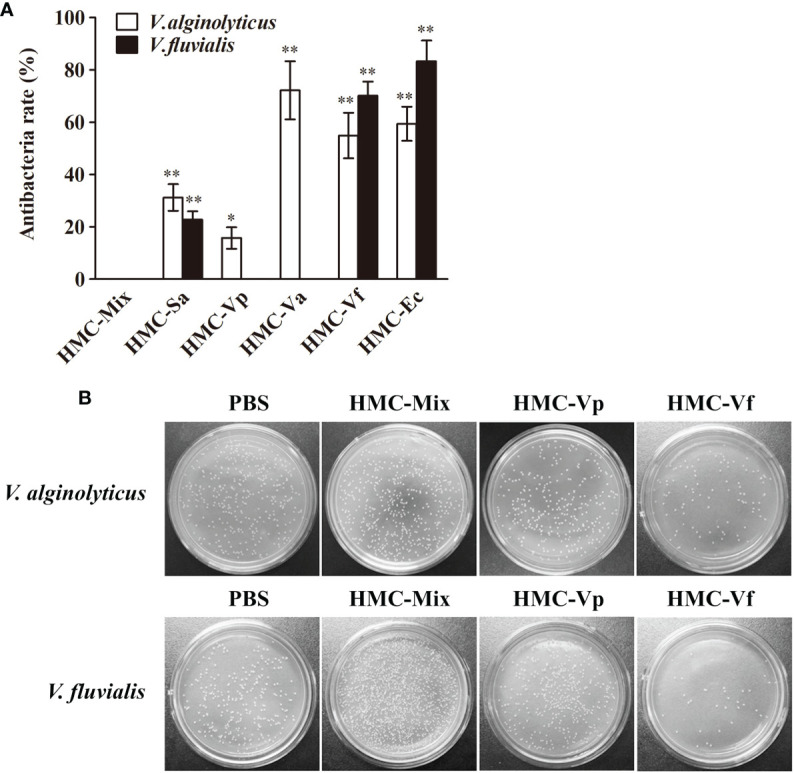
Antibacterial activities analysis of six bacteria-binding hemocyanin fractions from *L. vannamei*. **(A)** Comparison of antibacterial activities of 125 μg/ml HMC-Mix, HMC-Sa, HMC-Vp, HMC-Va, HMC-Vf, and HMC-Ec to *V. alginolyticus* and *V. fluvialis*, respectively. Data represent mean ± SD of at least three separate experiments. *p < 0.05, **p < 0.01. **(B)** Bacterial colonies of *V. alginolyticus* and *V. fluvialis* in Petri dishes treated with 50 μl HMC-Mix, HMC-Vp, HMC-Vf (125 μg/ml for each), respectively, 0.01 M pH 7.4 PBS as negative control.

Next, agglutinative and antibacterial activities were further performed to compare the immunological properties of the six HMC fractions. As shown in **Table 2**, agglutination could be observed when the six HMC fractions were separately bound with all of the five examined bacteria, showing agglutinative activities of 0.15–1.17 μg/ml. It is worth emphasizing that HMC-Sa, HMC-Vp, HMC-Va, HMC-Vf, and HMC-Ec possessed the strongest agglutinative activities against *S. agalactiae*, *V. parahaemolyticus*, *V. alginolyticus*, *V. fluvialis*, and *E. coli* K12, respectively, whose agglutinative titer was two- or fourfold as compared to the other four bacteria. Similarly, the six HMC fractions also showed a different degree of antibacterial activities against *V. alginolyticus* or *V. fluvialis*, respectively (**Figure 4A**). Higher antimicrobial activity was detected in HMC-Va against *V. alginolyticus*, and HMC-Vf and HMC-Ec against the two tested bacteria, showing inhibition of about 54.9–83.3%, but lower in HMC-Sa and HMC-Vp against *V. alginolyticus*. However, no activity was detected in HMC-Vp and HMC-Va to *V. fluvialis* and HMC-Mix against *V. alginolyticus* and *V. fluvialis* under the same conditions (**Figure 4A**). Similar results were found with counted colony method in **Figure 4B**. These results indicate that the specific bacterial resistance of HMC depends on co-effect of HMC isomers.

**Table 2:** Comparison of agglutinative activities of bacteria-binding hemocyanin with 75 μg/ml.

	HMC-Mix	HMC-Sa	HMC-Vp	HMC-Va	HMC-Vf	HMC-Ec
	Agglutinative titer^a^/agglutinative specific activity^b^					
*S. agalactiae*	256/0.29	256/0.29	512/0.15	256/0.29	64/1.17	256/0.29
*V. parahaemolyticus*	128/0.59	128/0.59	512/0.15	128/0.59	128/0.59	256/0.29
*V. alginolyticus*	128/0.59	128/0.59	256/0.29	512/0.15	64/1.17	256/0.29
*V. fluvialis*	256/0.29	128/0.59	128/0.59	256/0.29	256/0.29	512/0.15
*E. coli* K12	128/0.59	64/1.17	256/0.29	128/0.59	128/0.59	512/0.15

a The highest dilution of the testing samples in the presence of different bacteria.

b Agglutinative activity (μg/ml) = protein concentration (μg/ml)/agglutinative titer.

### 3.4 Glycosylation Modification Might Be Responsible for HMC Diversity

**Figure 5:**
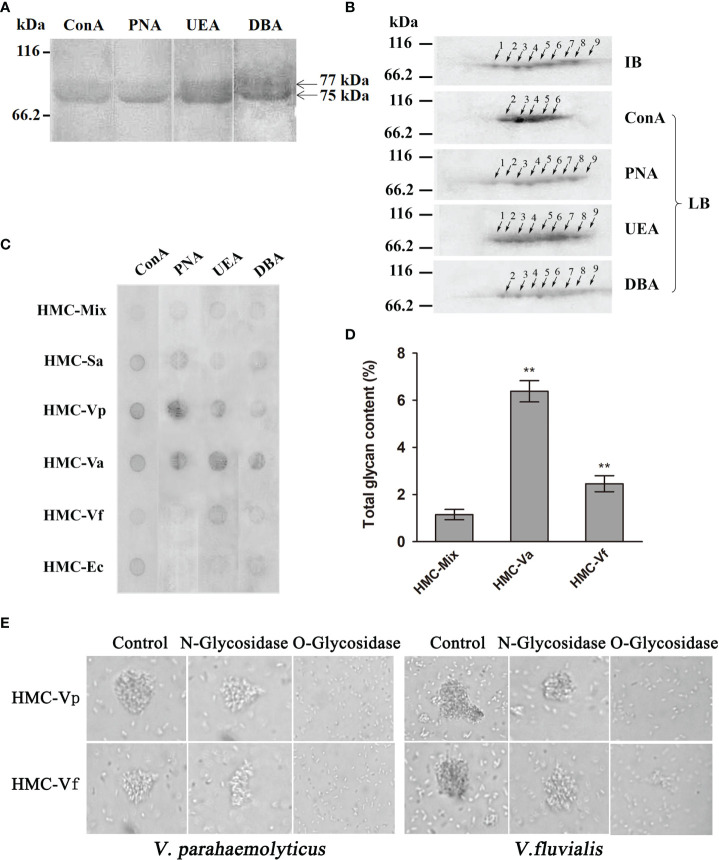
Analysis of glycosylation of hemocyanin from *L. vannamei*. **(A)** Lectin-blotting analysis of hemocyanin with ConA (1:1,000), PNA (1:100), UEA (1:500), and DBA (1:1000), respectively. **(B)** 2-D immunoblotting and 2-D lectin blotting with ConA, PNA, UEA, and DBA analysis of hemocyanin. IB, immunoblotting; LB, lectin blotting. Western blotting was performed using rabbit anti-shrimp HMC antisera (1:1,000 dilution) and goat anti-rabbit IgG-HRP (1:3,000 dilution) antibodies. Lectin-blotting analysis were carried out as the same descriptions with **(A)**. **(C)** Comparative analysis of the sugar content among six bacteria-binding hemocyanin by dot lectin blotting. ConA (1:20), PNA (1:5), UEA (1:5), DBA (1:5), and 50 μg/ml of HMC-Mix, HMC-Sa, HMC-Vp, HMC-Va, HMC-Vf, and HMC-Ec were used. **(D)** Comparison of sugar content of 200 μl of 250 μg/ml HMC-Mix, HMC-Va, and HMC-Vf by the method of phenol-sulfate acid. **(E)** Comparison of agglutinative activities of 50 μl HMC-Vp and HMC-Vf (300 μg/ml) treated with O-glycosidase or N-glycosidase against *V. parahaemolyticus* (left) or *V. fluvialis* (right) (2,000×), respectively. Untreated HMC-Vp and HMC-Vf were used as a control. **p < 0.01.

Since glycosylation is one of the most important post-translational modifications for immune molecules to regulate their function (39), we hypothesize that glycosylation difference may be also present in HMC isomers. To confirm the hypothesis, 1-D and 2-D lectin blotting was first applied. Lectins including concanavalin A (ConA, recognizing α-d-mannose > α-d-glucose), peanut agglutinin [PNA, recognizing β-D-gal-(1,3)-D- acetyl galactosamine], ulex europaeus agglutinin 1 (UEA, recognizing α-L-fucose), and dolichos biflorus agglutinin (DBA, recognizing *N*-acetyl-d-galactosamine) were used to blot the type of glycans in *L. vannamei* HMC purified by gel-filtration chromatography and anion-exchange chromatography. As expected, two bands at molecular weights approximately 75 and 77 kDa were observed, which could react with four lectins to different degrees (**Figure 5A**). For 2-D immunoblotting analysis, nine spots were detected (**Figure 5B**), and for 2-D lectin-blotting analysis, five to nine spots of HMC could bind specifically to the four examined lectins. Specifically, PNA, UEA, and DBA can recognize nine, nine, and eight HMC spots, respectively. However, ConA only recognized five HMC spots (**Figure 5B**). This indicates that different spots of HMC had different glycosylation modification. Next, dot lectin blotting and total glycan measurement were further performed to characterize glycosylation diversity of the above bacteria-binding HMC fractions and HMC-Mix as a control. As shown in **Figure 5C**, the six HMC fractions could bind to the four lectins, but the degree of binding was different. Among these, HMC-Va, HMC-Vp, HMC-Sa, HMC-Vf, and HMC-Ec could react with four lectins (ConA, PNA, UEA, and DBA), three lectins (ConA, PNA, and UEA), two lectins (ConA and PNA), one lectin (UEA), and one lectin (ConA), respectively, whereas HMC-Mix showed the lowest reaction to the four lectins compared with the other five HMC fractions. Consistently, the total glycan content of HMC-Va and HMC-Vf exhibited very significant difference (*p* < 0.01) compared with that of HMC-Mix. The HMC-Va carbohydrate content was 6.38%, which was about 2.6- and 5.4-fold to HMC-Vf and HMC-Mix (**Figure 5D**). These results suggest that glycosylation diversity might exist in HMC and HMC fractions bound to different bacteria.

To examine the relationship between immunological function and glycosylation modification of HMC, agglutination of *V. parahaemolyticus* and *V. fluvialis* was performed. We found that deglycosylated HMC fractions, dO-HMC-Vp and dO-HMC-Vf, by O-glycosidase, subsequently led to a general significant decrease in agglutinative activities to five kinds of pathogens, even completely abolished, whereas no effect was observed for those of deglycosylated HMC fractions, dN-HMC-Vp and dN-HMC-Vf, by N-glycosidase (**Table 3** and **Figure 5E**). These results suggest that O-linked carbohydrates play a crucial role in agglutinative activities to five kinds of pathogens.

**Table 3:** Comparison of agglutinative activity of two bacteria-binding hemocyanin and deglycosylation hemocyanin with 75 μg/ml.

	HMC-Vp	dN-HMC-Vp	dO-HMC-Vp	HMC-Vf	dN-HMC-Vf	dO-HMC-Vf
	Agglutinative titer^a^/Agglutinative specific activity^b^					
*S. agalactiae*	512/0.15	256/0.29	2/37.5	64/1.17	64/1.17	1/75
*V. parahaemolyticus*	512/0.15	256/0.29	1/75	128/0.59	64/1.17	1/75
*V. alginolyticus*	256/0.29	256/0.29	1/75	64/1.17	64/1.17	1/75
*V. fluvialis*	128/0.59	128/0.59	2/37.5	256/0.29	128/0.59	2/37.5
*E. coli* K12	256/0.29	128/0.59	2/37.5	128/0.59	128/0.59	1/75

a The highest dilution of the testing samples in the presence of different bacteria.

b Agglutinative activity (μg/ml) = Protein concentration (μg/ml)/agglutinative titer.

## 4 Discussion

Some non-specific immune molecules in invertebrates have been found to possess diversity at the protein level. Down syndrome cell adhesion molecule (Dscam) of *Drosophila* have the potential to express more than 18,000 isoforms of the immunoglobulin (Ig)-superfamily receptor (40). Similarly, the C-type lectin-like domain (CTLD) of *C. elegans* and fibrinogen-related proteins (FREPs) in the Pacific oyster *Crassostrea gigas* have diversity at nucleotide and protein levels (41, 42). Hemocyanin (HMC) is a large copper-containing respiratory protein found in the hemolymph of mollusks and arthropods. Recently, research revealed that HMC may be a novel and important non-specific innate immune defense molecule (2, 3). In crustaceans, HMC is thought to be composed of three distinct classes (α-type, β-type, or γ-type), although a single subunit is able to aggregate into hexameric structures (43). Within penaeid shrimp, β- and γ-type HMCs have been identified in *Litopenaeus vannamei* (33, 44–46). In this study, we identified at least nine protein spots as HMC small subunit (75 kDa) or large subunit (77 kDa). It is consistent with our previous research about small subunit HMC gene (47–49) and large subunit HMC gene (33, 34, 50), suggesting that HMC identified in this study belongs to γ-type and possesses significant HMC diversity at the protein level. Interestingly, other spots ranging from 20 to 60 kDa were also identified as HMC, which should be HMC fragments or modification isoforms (**Figure 1**). In combination with previous reports that HMC possessed polymorphisms including SNPs (30, 31) and alternative splicing variant (32) and could generate various antimicrobial peptides (7, 12, 17, 18, 51, 52) with resistance to pathogens infection, all these results indicated that HMC varied at nucleotide and protein levels.

Recently, many studies have indicated that HMC has multiple immune functions and could be involved in immune defense in invertebrate (2–26). Our previous publication displayed obvious molecular diversity including SNPs and variants at genomic and complementary DNA (cDNA) levels, which might contribute to the functional diversity of hemocyanin (30–34, 50). Some studies by other research groups also show that various hemocyanin isoforms were identified in *L. vannamei* (44–46). To further understand the responses of HMC isomers to pathogen challenge *in vivo*, the expression profile of the nine protein spots were examined; the detailed criteria are as follows: (1) the nine protein spots were detected repeatedly in different 2-DE gels; (2) the nine protein spots were identified as HMC subunit 75 or 77 kDa by MALDI-TOF-TOF-MS; (3) the molecular weights of the nine protein spot in the 2-DE gels were approximately 75 and 77 kDa, which is consistent with the two bands in 1-DE. After 6, 12, and 24 h of *S. agalactiae* and *V. parahaemolyticus* infection, spots 1–9 had different expressions (**Figure 2A**), indicating that HMC isoforms have different expression profile, which could be modulated by different bacteria. Similar results indicated that the expression of two hemocyanin subunits genes (PjHcL and PjHcY) from the shrimp *P. japonicus* could be strongly induced by WSSV infection, and PjHcL is more sensitive to WSSV infection than PjHcY (53). In black tiger shrimp *P. monodon*, 2D gels have identified several truncated HMC isoforms that were not only upregulated in response to bacterial infection but also showed *in vitro* antibacterial, antiviral, or agglutination activities (12, 51). Havanapan et al. reported that C-terminal HMC fragments were upregulated, whereas the N-terminal fragments were downregulated during Taura syndrome virus (TSV) infection in hemocytes of *P. vannamei* (54). This strongly suggested that the diversity of HMC is closely associated with its ability to recognize diverse pathogens.

Generally considered, the main mechanism of non-specific immune molecules recognizing pathogens was regarded as pathogen-associated molecular patterns (PAMPs), which are shared by these pathogens, representing conserved molecular patterns and absolutely essential for their physiology (29, 55). Interestingly, there is increasing evidence to support that several non-specific immune molecules can bind different pathogens and had the inhibitory property against bacteria (5, 56–59). C-type lectin, an important immune factor, might serve as LPS-specific pattern recognition receptor (PRR) to specifically recognize opportunistic bacterial and viral pathogens and thus play a role in the immune defense of aquatic shrimp *via* the binding and agglutination (56–58). Chitin-binding protein (CBP) from the kuruma shrimp *Marsupenaeus japonicus* could specifically recognize lipoteichoic acid, lipopolysaccharides, and peptidoglycans in the surface of several Gram-positive and Gram-negative bacteria, and facilitate the clearance of *V. anguillarum* (59). Notably, HMC directly bound to bacterial PAMPs, i.e., OmpT, OmpW, OmpX, OmpC, OmpA, and FadL, instead of intracellular and bacterial serine proteases and/or antimicrobial peptides. This binding can result in diverse biological actions including bacterial agglutination and growth inhibition, and human erythrocytes hemagglutination and hemolysis (5). However, the recognition mechanism of HMC to bacteria is not clear. In this study, immunological functions-based investigation showed that six kinds of pathogens-binding HMC fractions, namely, HMC-Sa, HMC-Vp, HMC-Va, HMC-Vf, HMC-Ec, and HMC-Mix, with diverse 2-DE profiles (**Figure 3B**), appeared to have different agglutinative and antibacterial activities against different bacteria. It is interesting to note that HMC-Sa showed higher agglutinative activities against Gram-positive bacteria *S. agalactiae* compared with Gram-negative bacteria, *V. alginolyticus*, and *V. fluvialis*. Similarly, high agglutinative and antimicrobial activities were detected in HMC-Va against *V. alginolyticus*, respectively (**Table 2** and **Figure 4**). These cumulative evidence for shrimp hemocyanin suggests that various HMC fractions involved in recognition of a specific microorganism may be a novel PRRs molecule to recognize pathogenic microorganisms in invertebrate.

We previously reported that HMC had an Ig-like conserved domain and could react with goat anti-human IgG, IgM, and IgA (5, 19, 24). Furthermore, we also found that the diversity of human IgG constituents and glycosylation levels may have functional significance (5, 36, 38). Therefore, to determine whether polymorphism of HMC glycosylation is associated with its functional diversity, glycan content and glycosylation level were investigated. The results showed that lectins binding varied markedly among the experimental groups (**Figure 5C**), and the total glycan content of HMC-Va and HMC-Vf exhibited significant difference (*p* < 0.01) compared with control (**Figure 5D**). Interestingly, deglycosylation of HMCs by O-glycosidase led to complete abolished agglutinative activities (**Table 3** and **Figure 5E**). In molluscan HMCs, the oligosaccharide structures of the structural subunits RvH2 from *Rapana venosa* hemocyanin (RvH), βc-*Helix lucorum* hemocyanin (βc-HlH), and *Megatura crenulata* keyhole limpet (KLH) reveal a complex N-glycan pattern combining typical structural features of different higher organisms; these glycosylation plays a crucial physiological role in the structural stability, immunostimulatory, and therapeutic effect of HMC (60, 61). Our previous research also found that *L. vannamei* HMC was deglycosylated using O-glycosidase; its agglutinative activity reduced about four- to eightfold (8, 38). The results from this study indicated that binding of different pathogens with HMC fractions showed diverse optimum immunological activities, and the differences in their immunological activities may be related to their glycosylation diversity. The remarkable diversity of hemocyanin glycan content is an important feature of their immune function and provides a new concept in pathogen–host interaction and is involved in a large number of biological recognition events in the host immune system. However, besides glycosylation diversity of HMC, other forms of diversity in protein level, such as degradation fragments and other post-translational modification of HMC, need further research.

In summary, the present study showed that HMC of *L. vannamei* possessed protein diversity. Furthermore, the polymorphism appeared to be closely associated with HMC’s recognition and resistance to diverse pathogens and interpretation of HMC with multiple immune activities. These results will contribute to an understanding of HMC’s diversity and its multifunctional mechanisms. Our findings may also be helpful to enrich and develop the knowledge of invertebrate’s immune system and immune prevention.

## Data Availability Statement

The original contributions presented in the study are included in the article/supplementary material. Further inquiries can be directed to the corresponding author.

## Author Contributions

XZ, JA, and YLZ wrote the manuscript. XZ, JQ, and YLZ conceptualized and designed the project. XZ, JA, XC, and YZZ performed data analysis. XZ, JQ, PZ, and ZZ performed experiments and collected samples. All authors contributed to the article and approved the submitted version.

## Funding

This work was sponsored by the National Natural Science Foundation of China (Nos. 31072237, 31872596, and 31502204), 2020 Li Ka Shing Foundation Cross-Disciplinary Research Grant (No. 2020LKSFG01E), and Key Special Project for Introduced Talents Team of Southern Marine Science and Engineering Guangdong Laboratory (Guangzhou) (No. GML2019ZD0606).

## Conflict of Interest

The authors declare that the research was conducted in the absence of any commercial or financial relationships that could be construed as a potential conflict of interest.

## Publisher’s Note

All claims expressed in this article are solely those of the authors and do not necessarily represent those of their affiliated organizations, or those of the publisher, the editors and the reviewers. Any product that may be evaluated in this article, or claim that may be made by its manufacturer, is not guaranteed or endorsed by the publisher.
